# Congenital toxoplasmosis and audiological outcome: from a case series to a suggestion of patient-based schedule

**DOI:** 10.3389/fped.2023.1297208

**Published:** 2024-01-04

**Authors:** S. Salomè, R. Malesci, V. Delle Cave, A. Amitrano, R. Gammella, F. Fanelli, E. Capone, L. Capasso, A. R. Fetoni, F. Raimondi

**Affiliations:** ^1^ Division of Neonatology, Department of Translational Medical Sciences, University of Naples “Federico II”, Naples, Italy; ^2^ Unit of Audiology, Department of Neurosciences, Reproductive and Odontostomatologic Sciences, University of Naples “Federico II”, Naples, Italy

**Keywords:** congenital toxoplasmosis, CT, audiologic assessment, SNHL (sensorineural hearing loss), audiological outcome

## Abstract

**Introduction:**

Sensorineural hearing loss (SNHL) has been suggested to be possibly related to congenital toxoplasmosis (CT), although its prevalence varies from 0% to 26%. This variance appears to be dependent especially on early timing of treatment. However, the available data are based on outdated studies conducted on small groups of patients that lack homogeneity. Therefore, to establish evidence-based guidelines for audiologic monitoring in CT, we conducted a comprehensive evaluation of a large case series over a long period of time.

**Patients and methods:**

This is a single-center, retrospective cohort that enrolled all infants and children who were exposed *in utero* to *Toxoplasma gondii* and/or congenitally infected between September 1980 and December 2022. They underwent standard serial audiological evaluations to detect possible SNHL at an early stage. The first evaluation was performed during the initial assessment to define the onset of congenital toxoplasmosis, with another evaluation conducted at least at 12 months of life.

**Results:**

We collected data from 1,712 patients, and 183 (10.7%) were diagnosed with CT. Among these cases, 78 children (42.6%) presented with symptomatic CT at the onset, exhibiting ocular findings (21.1%), clinical cerebral manifestations (6.1%), and/or abnormal findings on neuroimaging (35.5%). Therapy was administrated at the onset in 164 patients (89.6%) with 115 of them starting treatment prior to 2.5 months of age (0–388, median 32.00 ± 92.352 days of life). Only one patient presented with SNHL at the onset, but this was apparently unrelated to CT. The median number of audiological assessments was 2.2 ± 1.543 (2–10). No patients developed any grade of delayed hearing loss, both in treated and untreated groups. The median age at last audiological evaluation was 2.3 ± 2.18 years (1–8), although the median follow-up period was 12.4 years (±6.3), ranging from 1 to 27 years.

**Conclusions:**

Based on these data, it appears that SNHL may be less frequent in CT than previously assumed. We recommend conducting an audiological assessment at the onset (within the first 2.5 months of life) to comprehensively define the type of CT onset, and then conducting another evaluation within 9 months of life.

## Introduction

In the neonatal stage, congenital toxoplasmosis (CT) infection presented as asymptomatic in 85% of cases (1), although even children with no signs or symptoms at this age can develop lesions later in life, more frequently ocular lesions (2). Observational data describe that early treatment, both in pregnant women and newborns, results in good outcome with a normal neurological development (3, 4). By contrast, delayed therapy and/or unrecognized subclinical infection is related to increased risk of severe disabilities (3, 4). Prevalence of infection and severity of disease significantly differ worldwide (5, 6).

CT is a known risk factor for hearing loss (HL) (5) and parasites have been detected in the internal auditory canal, the spiral ligament, stria vascularis, and saccular macula in autopsy reports of the temporal bones by subjects with CT (6). These findings suggest that the hearing loss in these patients is directly mediated by *Toxoplasma gondii* and can result in sensory, neural, or sensorineural lesions (6). Hence, CT was considered as a risk factor for both congenital and delayed-onset hearing loss in the Joint Committee on Infant Hearing (JCIH) position paper (5).

In a systematic review by Brown et al. (7), the prevalence of toxoplasmosis-associated sensorineural hearing loss (SNHL) was described in 0%–26% of patients (8–13). This appears to depend especially on the timing of the treatment; in fact, it was rare in children appropriately treated in the first year of life. However, only five studies met the inclusion criteria indicating a lack of well-designed studies addressing the association between CT and SNHL. The study with the largest number of children and the longest follow-up (9) suggests that toxoplasmosis-associated SNHL may be rare in children adequately treated.

Considering that available data on the incidence of SNHL and the suggested audiological follow-up in patients with CT are based on few and ancient studies performed in small and inhomogeneous groups of patients, we decided to evaluate a large case series through a long-lasting follow-up to develop evidence-based guidelines for audiological monitoring in children following CT infection.

## Patients and methods

This is a single-center, retrospective cohort study conducted at the Specialized Perinatal Infection Unit of the University Federico II of Naples, in which all infants and children who were exposed *in utero* to *T. gondii* and/or congenitally infected between January 1980 and December 2022 were enrolled. The multidisciplinary team includes specialists from Neonatology, Maternal-Fetal Medicine, Pediatric Infectious Diseases, and Pediatric Audiologists dealing with the mother and infant dyad, focusing on vertically transmitted infections throughout the Campania Region. Serology examinations were performed by the local specialized reference laboratory.

Maternal infection was defined using Lebech's classification system and case definition (14). Data were collected regarding specific serological evaluations, time of diagnosis of toxoplasmosis, amniocentesis, and maternal therapy carried out in dosages and times. The inclusion criteria are indicated in the following.

Diagnosis of CT was made based on prenatal diagnosis (detection of *T. gondii* DNA in amniotic fluid), positive anti-*Toxoplasma* IgM and/or IgA at birth, a positive immunoblotting (performed in more recent years since the test was available), or an increase in IgG levels in the first months of life, along with specific clinical signs after a comprehensive clinical, radiological, and laboratory evaluation. Hence, the infants underwent a clinical and instrumental assessment of the central nervous system and a visual function evaluation. Moreover, accordingly with the targeted hearing screening program implemented in our institution, which became the Universal Newborn Hearing Screening (UNHS) program by 2007, all newborns were evaluated with auditory testing within the first month of life. Cranial ultrasonography (US) was performed by an experienced neonatologist, using the Philips HD11 ultra-sound imaging platform with 8.5–12.4 MHz transducers (Microconvex and Phased Array transducers). The fundus oculi examination was performed by a pediatric ophthalmologist skilled in congenital infections. The audiological assessment is detailed in the following.

Conversely, at the end of an adequate follow-up at 12 months, infants were considered only exposed *in utero* to *T. gondii* but not infected in the case of negative specific IgG, previously evidenced at birth. This population was described in detail in a previous manuscript (15).

The infected children were treated with varying doses of pyrimethamine depending on the severity of the disease at birth: daily doses of pyrimethamine (1 mg/kg) were administered for either 2 months (in asymptomatic children) or 6 months (in symptomatic children); thereafter this dose was received three times *per* week (i.e., on Mondays, Wednesdays, and Fridays) for the remaining 1 year; sulfadiazine (100 mg/kg/day, divided into two doses) was also administered for 12 months, and leucovorin was administered for 12 months as well (9). Treatment was started prior to 2.5 months of age and continued for 12 months because a shorter therapy can lead to severe disabilities (16). Before starting the therapy, the enzyme glucose-6-phosphate dehydrogenase (G6PD) was evaluated to rule out a deficiency that contraindicated the use of the drugs, and throughout the whole time of treatment patients were monitored clinically and serologically, to assess compliance, efficacy, and possible side reactions (17).

Therefore, the inclusion criteria for the study were as follows: being affected by CT and being exposed to *T. gondii in utero* but not infected; and the exclusion criteria included: parental denial to participate; infants who did not complete the 12-month follow-up; other congenital infectious potential causes of SNHL, such as a concomitant congenital cytomegalovirus (CMV) infection.

## Audiological assessment

The enrolled patients underwent periodic hearing evaluations to detect possible SNHL at an early stage. The auditory function was assessed by transient evoked otoacoustic emissions (TEOAE) at birth and by Automatic Auditory Brainstem Response (A-ABR) within the first month of life since 2013. Other evaluations were performed at least at the end of the first year of life and at 2 years of age. Before 2012, when the Italian recommendations on CT were published (18), children were evaluated annually until school age; after 2012, the last evaluation was performed at 2 years of age. In case of hearing loss, patients were examined more frequently, as determined by the Audiologist.

Different audiological tests were used to evaluate the children based on their age and were conducted in both ears in all cases. External and middle ear evaluations were performed in all enrolled subjects using otoscopy.

The audiological evaluation comprised the following: TEOAE, Visual Reinforcement Audiometry (performed from 6 months of age), Conditioned Play Audiometry (performed from 2 years of age), Acoustic immittance, and Brainstem Auditory Evoked Potential (BAEP) by click stimuli.

TEOAE and A-ABR tests were conducted either at birth centers or Pediatric Audiology Services by specifically trained personnel, while the infant was sleeping or following the end of feeding. The tests were recorded by the automated device Accuscreen Madsen newborn hearing screener (Natus, USA) whose output simply indicates the final response score (“pass” or “refer”). The TEOAE test was executed by paling the ear plugs in both ears, one ear at a time, and its evaluation was based on noise-weighted averaging counting of significant signal peaks; the stimuli were non-linear click sequences at 70 dB sound pressure level (SPL) with a frequency range of 1.5–4.5 kHz. The A-ABR test required both the ear plug in the ear and the montage of three electrodes with impedance kept ≤3,000 dines. The active/positive electrode was placed on the forehead, the exploring/negative electrode on the homolateral mastoid, and the mass/ground electrode, on the cheek. The clicks of A-ABR were delivered at a fixed intensity of 70 dB SPL. The device stops the recording as soon as the default “pass” criteria are met or after a given elapsed time. In the latter case, the response is scored as “refer.”

The diagnostic auditory brainstem responses (ABR) evaluation with threshold identification was performed by an audiometrist with a specific expertise in this field in one of the pediatric audiology services, in a sound proof and faradized room, during spontaneous sleep. The device used was Neuro-Audio, Inventis. The test was performed by standard skin preparation and the three electrodes montage with impedance kept ≤3,000 dines. One active electrode was applied on the forehead, one exploring electrode was placed on the homolateral mastoid, and one was a contralateral mass electrode. The standard procedure consists of alternate clicks at 21 pps, duration 0.1 ms, filter settings 100–2,000 Hz, and analysis time 12 ms. The protocol starts with a monaural stimulation at 80 dB nHL for the identification of the three main waves, I, III, and V—for the determinations of peak and inter-peak latencies. After this step, the stimulus is decreased at 10-dB steps up to a minimum of 20 dB nHL. Normal hearing was defined on the basis of presence and persistence of V wave for acoustic stimuli <30 dB nHL, while HL was defined as ≥30 dB nHL.

Various audiometric procedures (visually reinforced audiometry, conditioned play audiometry, and conventional audiometry) were adopted to identify the pure tone threshold using frequencies from 0.125 to 8 kHz with the device Resonance R37A (Resonance, Gazzaniga, Italy).

SNHL was defined as air conduction thresholds >20 dB, in conjunction with normal bone conduction threshold and normal middle ear function. Tympanometry with 226- and 1,000-Hz tone probes R36M (Resonance, Gaazzaniga, Italy) was performed for each child to confirm the diagnosis of SNHL. The criteria for normality in tympanometry included a sharp peak with middle ear pressure ranging from −150 to +25 daPa, static compliance between 0.2 and 0.9 ml, and ear canal volume within the normal range from 0.4 to 1 ml. Reduced or unmeasurable middle ear pressure along with a normal ear canal volume indicated the presence of middle ear dysfunction.

According to Bureau International for Audiophonology (BIAP) Classification 22, the degrees of HL were categorized as follows: normal (≤20 dB nHL), mild (21–40 dB nHL), moderate (41–70 dB nHL), severe (71–90 dB nHL), and profound (>91 dB nHL). We classified SNHL as monolateral if it was present in one ear or bilateral if it was present in both ears.

The audiological and clinical protocols are standardized in our institution, and the retrospective data were collected from medical records.

## Ethics approval and consent to participate

The Ethics Committee of our Institution (Comitato Etico “Carlo Romano”, Università Federico II di Napoli) approved this study (Protocol number 98/17). Consent to participate was obtained from parents or legal guardians of the children involved in the study.

## Data analysis

Data analysis was performed using SPSS v.28 software (IBM). Categorical variables were expressed as frequency (percentage) and quantitative variables as the mean (SD) or median (interquartile range, IQR) according to their distribution. Only descriptive statistics were used.

## Results

**Figure 1:**
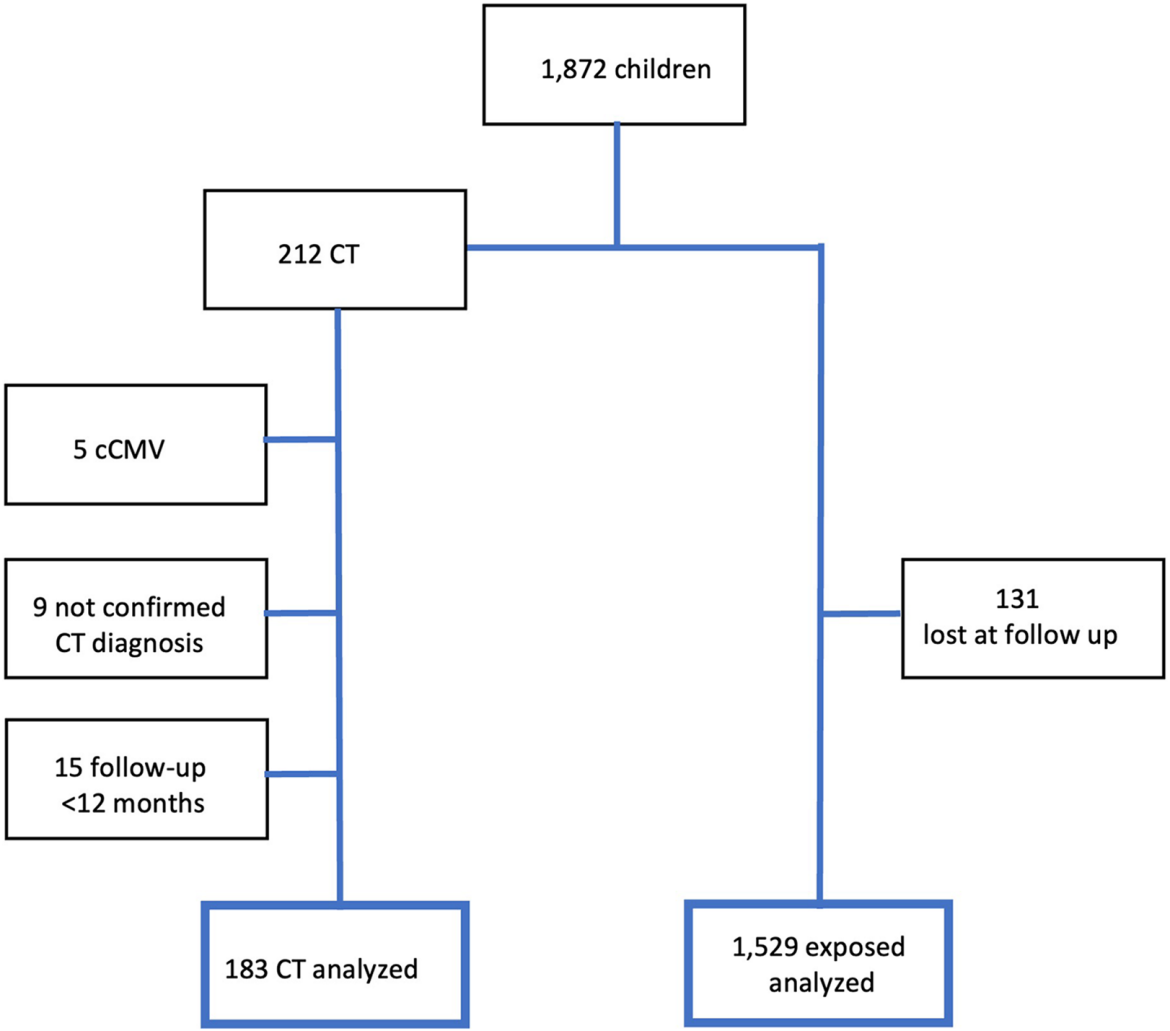
Study population.

Data on patients infected with CT are available since 1980, while data on those only exposed to *T. gondii* became available from 2010. Between 2010 and December 2022, we collected data from 1,872 children. After applying the established inclusion and exclusion criteria, 1,712 patients were selected for the present study. The flow chart of the study population is shown in Figure 1.

Of the 1,712 included patients, 183 (10.7%) were diagnosed with CT, while the remaining individuals were only exposed to infection during intrauterine life but not considered infected at the end of the evaluation period.

Among the infected patients, 122 (66.7%) were born before 2010, while the other 61 were born in or after 2010 (for which the data on exposed children are available). They were Italians in 94.5% of cases (173). Maternal age at delivery ranged from 16 to 42 years (median 30.00 ± 5.644). Seroconversion during pregnancy was demonstrated in 155 cases (84.7%). In most cases, the infection occurred in the third trimester (116 cases, 63.4%); with fewer occurrences in the second and first trimesters, accounting for 20.2% (37) and 5.5% (10), respectively. In 20 cases (10.9%), the timing of maternal infection could not be determined. A positive *T. gondii* DNA in amniotic fluid was detected in seven cases (3.8%), but amniocentesis was performed in 9.3% of women (17 cases). Maternal therapy was reported in 130 pregnant women (71%).

The gender distribution showed no difference, with 93 cases being male (50.8%). Data on pregnancy, birth, and early childhood were not available for 4 patients as they were adopted. Almost all but 11 (6%) were born premature with the gestational ages ranging from 32 to 42 weeks (media 38.59 ± 1.635). Birth weight was from 1,340 to 4,400 g (media 3,140 ± 503.097) and 33 (18%) children were small for their gestational age.

CT was asymptomatic at the onset in 101 children (55.2%), while the 78 symptomatic children (42.6%) presented with ocular findings (38, 21.1%), clinical cerebral manifestations (11, 6.1%), and/or abnormal findings on neuroimaging (64, 35.5%). The remaining four patients received a diagnosis of CT after the neonatal period because of recognition of chorioretinal scar in a routinely ophthalmological evaluation later on in life, so we could not define certainly the severity of their onset.

Antiparasitic therapy at the onset was administrated in 164 patients (89.6%) and in 115 of them (70.1%) treatment was initiated prior to 2.5 months of age. Specifically, therapy was started between 0 and 388 days of life (median 32.00 ± 92.352). There were 19 children who presented too late for treatment after an incidental diagnosis of non-active ocular lesion, so they were only periodically evaluated and treated if active lesions appeared.

Regarding audiological evaluation, only one patient presented with abnormalities at the onset. He was a male, born at term in 1995, small for his gestational age (birth weight 2,230 g) with microcephaly (head circumference 31 cm, ≤2DS). Seroconversion for *T. gondii* during pregnancy was retrospectively diagnosed, and the exact timing could not be determined because specific IgG and IgM were negative at 10 weeks of gestation, but they were not repeated and IgG were positive after delivery. The mother did not receive specific therapy. The newborn was diagnosed with CT because of positive IgM, IgG, IgA, and ISAGA IgM. Brain CT scan and ocular evaluation were normal. Therapy with Pyrimethamine and Sulfadiazine was started at 23 days of life and continued for one year without relevant sides effects. Neonatal hearing screening results were not available, but the first audiological evaluation was performed at 20 days of life with registration of TEOAE showing a bilateral refer result. At 2 months of age, click-evoked ABR and tympanometry showed a threshold of 60 dB nHL in the right ear and 70 dB nHL in the left ear. ABRs performed during follow-up confirmed bilateral SNHL. Pure tone audiometry showed symmetrical moderate high-frequency SNHL, and the speech recognition score was 100% for both ears using phonetically balanced words at 60 dB nHL bilaterally. The patient was a candidate for conventional amplification hearing aids, but his parents refused them, as he did himself when he grew up. With regard to the family history, the father and the paternal grandfather had SNHL. Given the family history of SNHL, he underwent genetic investigation for mutations in the connexin genes, which proved negative for *GJB2* and *GJB6*. Comprehensive genetic testing using massively parallel sequencing or next-generation sequencing were not performed owing to the family's refusal. Brain magnetic resonance imaging (MRI) showed no intracranial lesions and abnormalities of the internal auditory meatus or cerebellum pontine angles. He exhibited mild language delay, particularly with regard to phonological skills, but his neurodevelopment was otherwise normal. He was evaluated until the age of 20 years old and did not develop chorioretinitis.

The median number of audiological assessments considered was 2.2 ± 1.543 (range: 2–10). The assessments showing middle ear disorders causing transient conductive hearing loss were excluded. None of the patients developed any grade of delayed hearing loss, either in the treated or the untreated groups. The median age at the last audiological evaluation was 2.3 ± 2.18 years (range: 1–8), although the median follow-up period was of 12.4 years (±6.3), ranging from 1 to 27 years.

Among the 1,529 exposed patients, three (2‰) presented with bilateral SNHL at the first audiological evaluation. Congenital CMV infection was excluded. One was lost at follow-up, another presented with SNHL in a more complex syndromic picture, and the third carried a novel mutation in the GJB2 gene.

## Discussion

Congenital hearing loss is one of the most prevalent chronic conditions in children with a rate among newborns of one to three per 1,000 live births (19). It is commonly attributed to environmental factors such as congenital infections, including toxoplasmosis (20), as well as genetic factors, encompassing both non-syndromic forms and syndromes. Furthermore, congenital infections are known risk factors for delayed/progressive and acquired hearing loss (21, 22). Currently available data on SNHL in CT were collected from a small population (7). In Table 1, our data are compared with those reported in the review by Brown et al. (7), the study of Macedo de Resende et al. (13), and to the one of Auriti et al. (23), which are more recent. We did not include data by Austeng et al. (24) because they found no cases of hearing loss in the offspring of 40 women with primary *T. gondii* infection in pregnancy, but they did not report if the children described were infected or only exposed *in utero*. Moreover, data by McGee et al. (25) were included in the study by McAuley et al. (10).

**Table 1:** Overview of the audiological outcome in CT.

Study	Cases	Duration of follow-up	Age at start of therapy	Clinical manifestations	Number of audiological assessments	Age of last audiological assessment	Number of patients who developed SNHL	Hearing aids/language deficit
Salomè et al. (2023), Italy	183	12.4 ± 6.3 (1–27) years median	32.00 ± 92.352 (0–388 days) median	78 symptomatic patients: 38 ocular findings, 11 clinical cerebral manifestations, 64 abnormal findings on neuroimaging	2.2 ± 1.543 (1–10) median	2.3 ± 2.2 (1–8) years median	0	0
Auriti et al. (23), Italy	18	Up to 2–4 years of life	NA	Six symptomatic at birth	NA	NA	0	5/14 (35.7%) language delay
Macedo de Resende et al. (13), Brazil	106	NA	<2.5 months (50 days)	84 eye pathology	NA	24 months	Four (3.8%)	28 patients (two of them with SNHL)
de Andrade et al. (8), Brazil	19	3 years	NA	Four with hepatosplenomegaly (two hydrocephaly and microphthalmia)	19 cases OAE 17 cases ABR	624 days (1.7 years) median value	Four (21.1%) (one severe, one moderate, two mild–moderate)	Two cases were referred to wear hearing aids and undergo speech and hearing therapy
McLeod et al. (9), mainly USA and Canada	120	10.5 ± 4.8 (0.2–21) years mean	<2.5 months	12 nothing 12 mild 96 severe (neurological sequelae and chorioretinitis)	NA	NA	0	0
McAuley et al. (10), mainly USA and Canada	44	15 years	<2.5 months	20 hepatosplenomegaly 25 CNS calcifications 30 retinal lesions	NA	NA	0	0
Wilson et al. (11), USA	24	8.5 years (average age at last evaluation)	– Nine infants treated for at least 3 weeks before the age of 1 year; – Four treated only after developing sequelae or for less than 2 weeks; – 11 no treatment	– 22 chorioretinitis – Six major neurologic sequelae; – Five intracranial calcifications	NA	NA	Five (26%): – 2/19 moderate unilateral; – 1/19 mild unilateral; – 2/19 mild bilateral	NA
Stagno et al. (12), USA	12	1–86 months	Nine infants for at least 3 weeks before the age of 1 year	Nine eye pathology: (nine chorioretinitis, six nystagmus, four optic atrophy, seven strabismus)	Average of 2.3 examinations per patient	NA	0 of 7 tested	0

NA, not available; CNS, central nervous system.

In our cohort of patients with CT, only one patient presented with sensorineural hearing loss that did not appear to be related to CT. In fact, this patient had an asymptomatic onset of CT and developed no signs or symptoms related to this infection until the age of 20. Although genetic tests available at the time were negative (no mutations in the connexin genes), there was a positive family history of SNHL in the paternal line, with both the father and paternal grandfather having SNHL. However, the patient's family opted not to undergo next-generation testing. In our only exposed children, we identified three patients with SNHL representing a prevalence of two in 1,000 newborns, consistent with what has been described in the general population (19).

A detailed evaluation of the children with SNHL described by Wilson et al. (11) did not reveal whether they received appropriate treatment or other characteristics that could better define this group. Out of the four children with sensorineural impairment described by de Andrade et al. (8), the only patient with significant functional loss was severely affected by the infection at birth and presented other risk factors for hearing loss besides parasitosis (low birth weight and long-term use of assisted ventilation). This patient developed hearing and ocular sequelae despite treatment. Another child who was properly treated still experienced hearing loss, suggesting that CT, common in Brazil, may be a risk factor for hearing impairment and its impact on hearing loss requires further studies (13). Patients described by Macedo de Resende et al. (13) initiated treatment before 2.5 months of age, but the authors hypothesized the possibility of increased parasite virulence and/or higher individual susceptibility in the studied region (Brazil). However, their preliminary results needed confirmation with a larger sample and a longer follow-up.

As shown in the table, our series is larger than the previously described ones. Moreover, the follow-up lasted until 27 years of age with a median of 12.4 ± 6.3 years, which is longer than in other studies. The audiological follow-up was longer as well. In this large and long-term series, no patients presented with SNHL related to CT at the onset, confirming previous preliminary data [Stagno et al. (12), McAuley et al. (10), McLeod et al. (9), and Auriti et al. (23)]. In the case series by Auriti et al. (23), only one out of 18 infected infants (5.6%) had a mild SNHL at 12 months that was not confirmed at both the 2- and 4-year follow-up visits. They also reported isolated language delay (i.e., without concomitant SNHL) in 35.7% of their patients. In the cohort described by Macedo de Resende et al., a significant language delay was present in 28/106 patients (26.4%). As two children did not have an associated SNHL, the authors suggested that their language delay was part of a wider neurodevelopmental impairment. An isolated language delay was not observed in our large cohort.

Possible audiological damage remains plausible based on the pathophysiology of the infection (6). Therefore, we continue to recommend at least one audiological assessment at the onset in CT patients, as early diagnosis allows the implementation of therapeutic support, preventing the development of speech and psychomotor delays. Furthermore, no patient developed sensorineural hearing loss related to CT, even when treatment was initiated after 2.5 months.

Brown et al. (7) suggested that children with CT who have received a 12-month course of antiparasitic therapy initiated before 2.5 months with serologically confirmed compliance should have repeat audiometric evaluation at 24–30 months of age until further evidence was available. They also recommended that children with CT that received no treatment, partial treatment, delayed onset of treatment, or presented low compliance should undergo annual audiological monitoring until they are able to reliably report hearing loss. Our data provide the desired missing evidence cited by Brown et al. (7) and suggests that the previously proposed annual audiological monitoring (7, 26) may be unnecessary and without benefits, both in terms of childhood development and financial implications. Moreover, prolonged audiological follow-up is not beneficial for patients and can be a source of stress for them and their family. Our data support the generic recommendation of the JCIH (5), recently confirmed by American Academy of Pediatrics (27), for infants who passed newborn hearing screening but had *in utero* infections, requiring follow-up by 9 months of age. It should be advisable to always use ABR when testing CT children because, if a risk is possible, it involves the central hearing (28). Correlations between CT and a high prevalence of hearing problems and language delay have been described in Brazil (Congenital Toxoplasmosis Brazilian Group) where this disease is more frequent and severe than in Europe (13). So, we suggest this schedule only for Europe and areas with similar epidemiology for *T. gondii*.

Finally, in children with a CT diagnosis that is completely asymptomatic at the onset except for SNHL, it is essential to evaluate other etiologies such as congenital CMV infection or genetic diseases.

## Conclusions

The follow-up of CT infection is mainly dedicated to the ophthalmological, neurological, auditory, and serological aspects. What was particularly useful was the demonstration of the lack of the necessity of many audiological assessments. In fact, this paper tried to support a more rational schedule of follow-up to not miss SNHL diagnosis but also to avoid useless stress for patients and families and to better organize medical resources. In fact, based on our data and on the literature review, it seems prudent to suggest an audiological assessment at the onset (within the first 2.5 months of life) to comprehensively define the type of onset of the congenital toxoplasmosis, investigating all target organs, so that if the patient is deaf, the right process can begin early, and a new evaluation within 9 months of life.

## Data Availability

The datasets presented in this paper are not readily available because data are not anonymized. Requests to access the datasets should be directed to serena.salome@unina.it.
